# Surveillance of dengue vectors using spatio-temporal Bayesian modeling

**DOI:** 10.1186/s12911-015-0219-6

**Published:** 2015-11-13

**Authors:** Ana Carolina C. Costa, Cláudia T. Codeço, Nildimar A. Honório, Gláucio R. Pereira, Carmen Fátima N. Pinheiro, Aline A. Nobre

**Affiliations:** 1 Sergio Arouca National School of Public Health, Oswaldo Cruz Foundation, Rua Leopoldo Bulhões 1.480, Rio de Janeiro, Brazil; 2 National Institute of Women, Children and Adolescents Health Fernandes Figueira, Department of Clinical Research Oswaldo Cruz Foundation, Avenida Rui Barbosa 716, Rio de Janeiro, Brazil; 3 Scientific Computing Program, Oswaldo Cruz Foundation, Avenida Brasil 4365, Rio de Janeiro, Brazil; 4 Laboratory of Transmitters of Hematozoa, Oswaldo Cruz Institute, Oswaldo Cruz Foundation, Avenida Brasil 4365, Rio de Janeiro, Brazil; 5 Sentinel Operational Unit of Mosquito Vectors, Oswaldo Cruz Foundation, Avenida Brasil 4365, Rio de Janeiro, Brazil

**Keywords:** Entomological surveillance, Dengue, Bayesian methods, Spatio-temporal models, Zero-inflated models, INLA

## Abstract

**Background:**

At present, dengue control focuses on reducing the density of the primary vector for the disease, *Aedes aegypti*, which is the only vulnerable link in the chain of transmission. The use of new approaches for dengue entomological surveillance is extremely important, since present methods are inefficient. With this in mind, the present study seeks to analyze the spatio-temporal dynamics of *A. aegypti* infestation with oviposition traps, using efficient computational methods. These methods will allow for the implementation of the proposed model and methodology into surveillance and monitoring systems.

**Methods:**

The study area includes a region in the municipality of Rio de Janeiro, characterized by high population density, precarious domicile construction, and a general lack of infrastructure around it. Two hundred and forty traps were distributed in eight different sentinel areas, in order to continually monitor immature *Aedes aegypti* and *Aedes albopictus* mosquitoes. Collections were done weekly between November 2010 and August 2012. The relationship between egg number and climate and environmental variables was considered and evaluated through Bayesian zero-inflated spatio-temporal models. Parametric inference was performed using the Integrated Nested Laplace Approximation (INLA) method.

**Results:**

Infestation indexes indicated that ovipositing occurred during the entirety of the study period. The distance between each trap and the nearest boundary of the study area, minimum temperature and accumulated rainfall were all significantly related to the number of eggs present in the traps. Adjusting for the interaction between temperature and rainfall led to a more informative surveillance model, as such thresholds offer empirical information about the favorable climatic conditions for vector reproduction. Data were characterized by moderate time (0.29 – 0.43) and spatial (21.23 – 34.19 m) dependencies. The models also identified spatial patterns consistent with human population density in all sentinel areas. The results suggest the need for weekly surveillance in the study area, using traps allocated between 18 and 24 m, in order to understand the dengue vector dynamics.

**Conclusions:**

*Aedes aegypti*, due to it short generation time and strong response to climate triggers, tend to show an eruptive dynamics that is difficult to predict and understand through just temporal or spatial models. The proposed methodology allowed for the rapid and efficient implementation of spatio-temporal models that considered zero-inflation and the interaction between climate variables and patterns in oviposition, in such a way that the final model parameters contribute to the identification of priority areas for entomological surveillance.

**Electronic supplementary material:**

The online version of this article (doi:10.1186/s12911-015-0219-6) contains supplementary material, which is available to authorized users.

## Background

The complexity of dengue transmission has motivated the development of many studies to assess the numerous factors related to the circulation and persistence of this disease in human populations. While significant progress has been made, numerous questions remain unanswered, and an effective dengue control plan is still an open problem. No effective vaccines or antiviral drugs are currently available, impeding a direct intervention on the human link in the dengue transmission cycle. As such, current control measures are still focused on reducing the vectorial capacity of *Aedes aegypti*.

The Household Infestation index (HI) and the Breteau index (BI) are the standard mosquito abundance measurements used to evaluate the effectiveness of vector control strategies [1]. However, these indices, based on the survey of breeding sites with mosquito larvae, are poorly correlated with the abundance of the adult mosquito population, which is directly responsible for the disease transmission [2]. For example, a study has shown high dengue incidence when HI was below 3 % in Salvador city, Northeastern Brazil [3].

Ovitrap has been proposed as an alternative tool for *Aedes aegypti* monitoring in areas with low mosquito abundance [4]. This trap was created by Fay and Eliason [5] and perfected by Reiter and Gubler [6], and provides measures of ovipositing activity. Although not a direct measure of mosquito abundance, studies have shown a strong correlation between egg count and the density of the female mosquito population [7].

Despite the advantages of trapping over larval surveys, this approach is still scarcely applied to surveillance. One of the main reasons for this is the fact that the sampling and statistical properties of the measurements produced are not entirely understood yet. In this study, data from a long-term surveillance program carried out under very controlled conditions in eight sentinel areas, provide an opportunity to develop and test innovative models and contribute to the development of an analytical framework to be implemented in *Aedes aegypti* monitoring systems. The models proposed are computationally intensive and more efficient methods that allow for their implementation in surveillance and monitoring systems were investigated. Such information contributes to the development of *Aedes* control activities focused on areas and time periods affected by the most severe mosquito infestations.

## Methods

### Study area

**Fig. 1:**
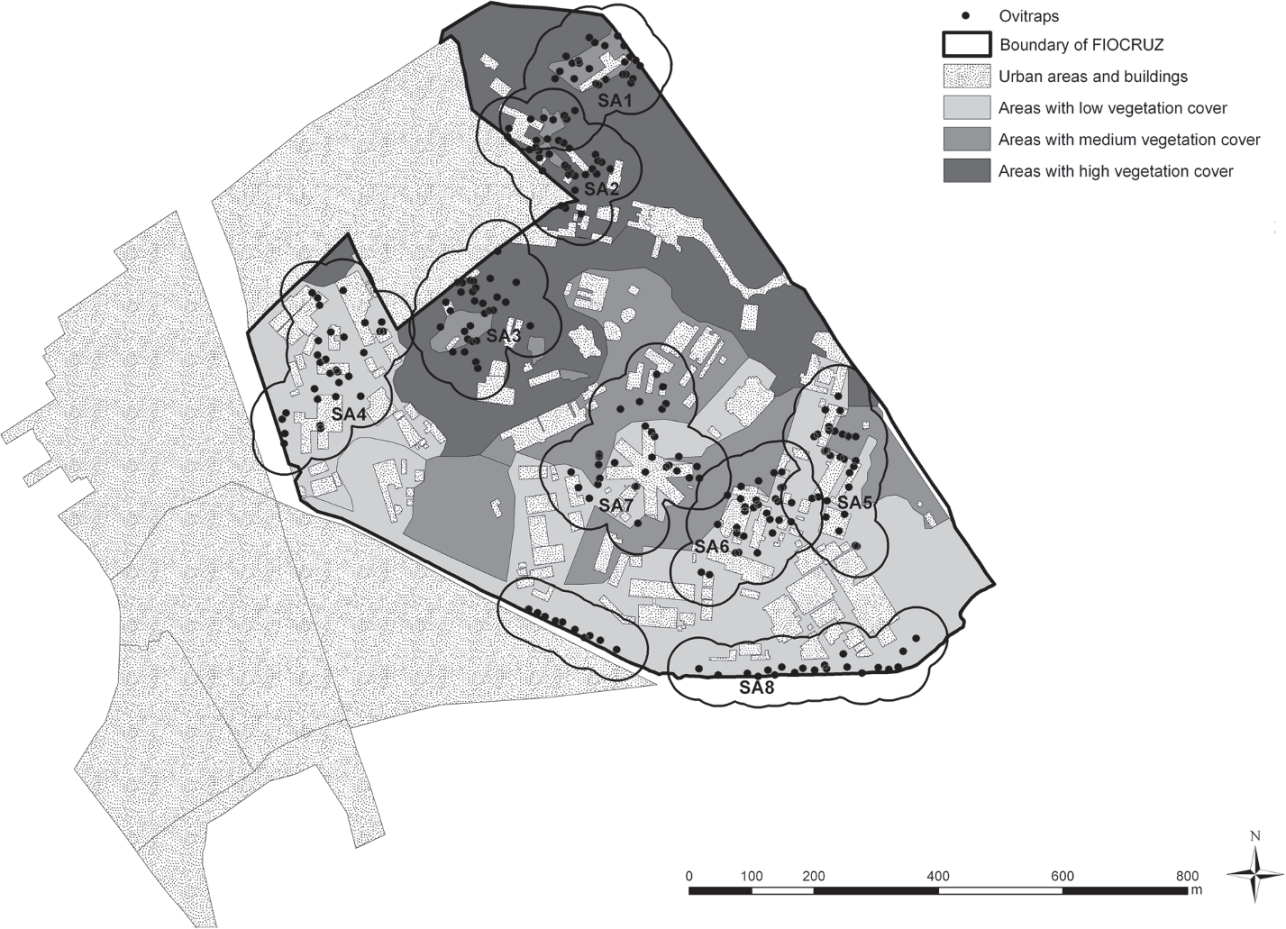
Map of Manguinhos campus at Oswaldo Cruz Foundation. Characterization of green space, and localization of oviposition traps in eight sentinel areas (SA). The SAs were located at different altitudes, in such a way that none of the habitats overlapped

The study area is the Manguinhos campus of the Oswaldo Cruz Foundation (Fiocruz), located in the city of Rio de Janeiro, Brazil (22°52’30”S, 43°14’53”W; 697.000 *m*^2^). The area surrounding the campus is characterized by densely populated urban zones, precarious living conditions and a general lack of infrastructure [8]. Eight sentinel areas were identified for continuous monitoring of immature *Aedes aegypti* and *Aedes albopictus*, each one representing different degrees of forest cover, distances to neighbouring residential areas, and intensity of human commutation and permanence. Sentinel Areas (SA) were designated as SA1–SA8 (Fig. 1). Table 1 describes the area and the vegetation-type percentage for each SA.

**Table 1:** Descriptors

Sentinel Area	Area	Vegetation type (%)				
(SA)	(*m*^2^)	Urban	Low	Medium	Dense	Total
SA1	42,701.18	8	0	16	59	83
SA2	41,388.24	35	0	5	61	100
SA3	46,051.25	26	0	10	64	100
SA4	57,714.65	36	51	0	5	92
SA5	43,686.54	25	53	18	4	100
SA6	43,515.28	28	47	25	0	100
SA7	61,048.08	24	26	50	0	100
SA8	71,055.57	18	53	0	0	71

Area and vegetation cover in each SA at the Fiocruz Manguinhos campus, Rio de Janeiro

### Entomological monitoring

Thirty ovitraps were randomly placed in each SA with a minimum distance of 20 m between traps. Traps consisted of black plastic pots containing water, hay infusion and a eucatex paddle. Egg collections occurred weekly from November 2010 to August 2012, for a total of 89 uninterrupted weeks. The collected material was transported to the Sentinel Operational Unit of Mosquito Vectors (NOSMOVE/Fiocruz). Each paddle was carefully inspected for egg positivity and, when confirmed, the number of eggs was quantified. The sampling using ovitraps is part of the project “Monitoring of populations of *Aedes aegypti* on the Fiocruz campus” which is coordinated by Dr. Honório and was approved by the Vice-Presidency of Environment, Healthcare and Health Promotion (VPAAPS) of Fiocruz.

### Climate and environmental variables

Maximum and minimum temperatures were collected from the São Cristóvão meteorological station, located at a 3 km-distance from the study area, while accumulated rainfall was extracted from the Penha station, roughly 5 km away (source: Sistema Alerta Rio website [9], operated by the city of Rio de Janeiro).

To delimit the area of each SA, 50 m-radius influence zones were created for each trap, based its geographical location, using ArcGis 10.0 software. In order to calculate the percent green cover for each of the eight areas, a campus map was intersected with the oviposition traps by vectorizing a high-resolution satellite image. Finally, the distance between each trap and the nearest boundary of the study area was calculated and is hereby referred to as Border Distance.

### Spatio-temporal modeling

**Fig. 2:**
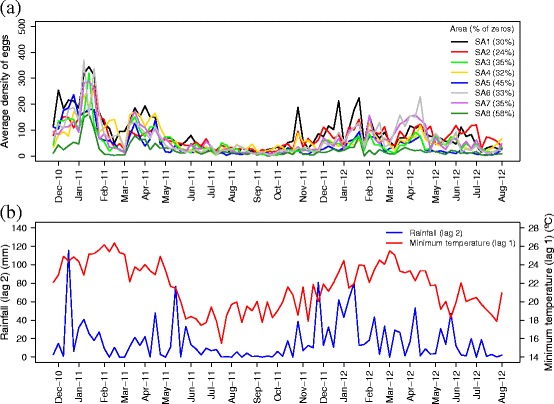
Temporal Series. Time series of the average weekly egg density and the zero-egg collection week frequency in each of the eight SAs (**a**), accumulated rainfall (lag 2) and minimum temperature (lag 1) (**b**)

Figure 2a shows weekly average egg density in each of the eight sentinel areas, together with zero-egg frequency, that varied from 24 to 58 %. Ignoring zero-inflation leads to two possible consequences: 1) biased estimation of model parameters and standard errors, and 2) overdispersion. Due to the high frequency of zeros, a zero-inflated model was considered. The modeling approach was: first, use a Binomial (Bin) distribution to model the zero occurrence probability. Then, model the non-null observations using a Zero-Altered Poisson (ZAP) distribution [10]. The underlying assumption is that two separate ecological processes are occurring: presence of eggs is driven by the mosquito choice of the ovitrap for ovipositing, and the abundance of eggs is driven by the number of females that chose the ovitrap. This can be formalized as following:

$$\begin{array}{@{}rcl@{}} \begin{array}{c} f_{ZAP}(y;\zeta,\mu)=\left\{ \begin{array}{l} 1-\zeta \;\qquad\qquad\qquad\quad\quad\quad\quad\;\; y=0\\ \zeta \times f_{ZAP}\left(y;\mu\right)\;\quad\quad\quad\quad\quad\quad\;\; y>0, \end{array} \right. \end{array} \end{array} $$

in which 1−*ζ* represents the probability of the absence of oviposition. Analogously, *ζ* is the probability of the occurrence of oviposition and will be referred to as positivity.

Let the random variable *Y*(*s*_*i*_,*t*) represents the number of eggs in trap *i*, *i*=1,…,30 at week *t*, for *t*=1,…,89. Moreover, let *y*(*s*_*i*_,*t*) be the realization of the spatio-temporal process *Y*(*s*_*i*_,*t*), when oviposition occurs. It is assumed that *y*(*s*_*i*_,*t*) has a ZAP distribution with an average of *μ*(*s*_*i*_,*t*) and the following equations:

(1) $$\begin{array}{@{}rcl@{}} y(s_{i},t) \mid y(s_{i},t) > 0 &\sim & ZAP(\mu(s_{i},t)) \end{array} $$

(2) $$\begin{array}{@{}rcl@{}} log(\mu(s_{i},t))&=&z(s_{i},t)\beta+\xi(s_{i},t)+\varepsilon(s_{i},t), \end{array} $$

(3) $$\begin{array}{@{}rcl@{}} \xi(s_{i},t)&=&a\xi(s_{i},t-1)+\omega(s_{i},t) \end{array} $$

for *t*>1, where *z*(*s*_*i*_,*t*)=(*z*_1_(*s*_*i*_,*t*),…,*z*_*p*_(*s*_*i*_,*t*)) denotes the vector of *p* covariates for trap *i* in time *t*, and *β*=(*β*_1_,…,*β*_*p*_)^′^ is the vector of coefficients representing their effects. Additionally, $\varepsilon (s_{i},t) \sim N\left (0,\sigma ^{2}_{\varepsilon }\right)$ is the measurement error defined by a Gaussian white noise, both serially and spatially uncorrelated. In the geostatistics literature, the term *z*(*s*_*i*_,*t*)*β* is referred to as the large-scale component – in this case depending on meteorological and environmental covariates – while the variance $\sigma ^{2}_{\varepsilon }$ is called the nugget effect [11]. Finally, *ξ*(*s*_*i*_,*t*) is a space-time Gaussian Field (GF) that follows an auto-regressive first-order dynamics, with temporal correlation coefficient *a* and evolution error given by *ω*(*s*_*i*_,*t*), in which |*a*| <1 and *ξ*(*s*_*i*_,1) derived from the stationary distribution $N\left (0,\sigma ^{2}_{\omega }/(1-a^{2})\right)$. Additionally, *ω*(*s*_*i*_,*t*) has a Gaussian distribution with zero average, no time dependence and characterized by the following covariance function

$$\begin{array}{@{}rcl@{}} \text{Cov}(\omega(s_{i},t),\omega(s_{j},t^{\prime})) = \left \{ \begin{array}{ll} 0 &\text{if}\; t \neq t^{\prime}\\ \sigma^{2}_{\omega} \mathcal{C}(h) &\text{if}\; t=t^{\prime}\;, \end{array} \right. \end{array} $$

for *i*≠*j*. The spatial correlation function $\mathcal {C}(h)$ depends on the spatial Euclidean distance between locations *s*_*i*_ and *s*_*j*_, such that $h=\,\mid \mid s_{i} - s_{j} \mid \mid \;\in \mathbb {R}$. This way, the process is assumed to be second-order stationary and isotropic [11]. It follows immediately that $Var(\omega (s_{i},t))=\sigma ^{2}_{\omega }$, for each *s*_*i*_ and *t*. The spatial correlation function $\mathcal {C}(h)$ is defined by the Matérn covariance function

(4) $$\begin{array}{@{}rcl@{}} \mathcal{C}(h)=\frac{1}{\Gamma(\nu)2^{\nu-1}}(\kappa h)^{\nu} K_{\nu}(\kappa h),  \end{array} $$

with *K*_*ν*_ denoting the modified Bessel function of the second type and order *ν*>0. The parameter *ν*, which is usually kept fixed, measures the smoothness of the process. In other words, *ν* controls the behavior of the covariance function for measures that are separated by small distances. On the other hand, *κ*>0 is a scaling parameter related to range *ρ*, i.e., a distance at which spatial correlation becomes small. In particular, we use the empirically derived definition $\rho =\frac {\sqrt {8\nu }}{\kappa }$, with *ρ* corresponding to the distance at which spatial correlation is approximately 0.1, for each *ν* (see Lindgren et al. [12] for further details).

In order to identify the climate and environmental covariates that best predicted the number of eggs, ZAP models were adjusted by area for each of the following climate variables: accumulated rainfall, minimum and maximum temperature. As the effect of these variables may not be immediately related to egg density, three-week lag periods were investigated for each variable and the best lag chosen using biological plausibility and Deviance Information Criteria (DIC) [13] available in the R-INLA package [14]. Then, each selected climate variable was tested for interaction with every other climate variable in the model. Figure 2b shows the climate variables composing the model. Besides the aforementioned variables, the only environmental covariate considered for inclusion in the spatio-temporal model was border distance. Because the variable-measuring scales were different, each covariate was standardized by subtracting the mean and dividing it by the standard deviation.

**Fig. 3:**
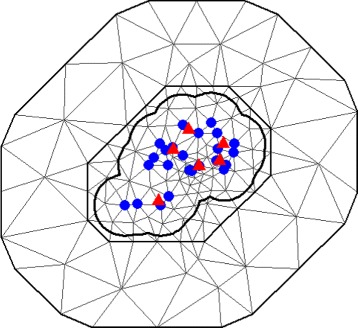
Triangulation. Locations of the 30 oviposition traps in the sentinel area 1 (SA1) and triangulation of SA1 with 147 vertices. Blue circles identify the traps used to estimate model parameters and red triangles identify those used for validation of the proposed model

We used 24 traps for model fitting (blue dots in Fig. 3) and the remaining six traps to validate the model (red triangles in Fig. 3). The predictive performance of the models was evaluated by calculating the percentage of observations, which fell within the 95 % credibility intervals for validating data.

In addition to the adjustment of individual models per SA, a hierarchical model was also considered for the whole set of sentinel areas.

#### Hierarchical model

This model is similar to the one presented above, but includes an area-specific random effect. In this analysis, from the total of 240 traps, 192 traps were used for model fitting and 48 ones for validation. As before, the effect of the interaction among climate variables was also tested. The validity of the model was checked as before.

### SPDE approach

***ξ*** is a spatially structured GF pursuant to the effect of ***ω***, in such a way that it can be considered to be a multivariate Normal distribution. $\tilde {\Sigma }$ is the dense correlation matrix, which describes the covariance structure of ***ξ***. The factorization of $\tilde {\Sigma }$ has a computational cost of the order *O*(*n*^3^), which can pose a problem in case of large array. Thus, the suggestion is to represent the GF as a Gaussian Markov Random Field (GMRF) based on Stochastic Partial Differential Equations (SPDE) [15].

The GMRF is a process that models the spatial dependence of the data by areal unit, such as regular/irregular grids, or by geographic region. The primary advantage of using GMRF instead of GF entails its strong computational properties. The computational advantage of making inference with GMRF stems directly from the sparsity of the precision matrix $\tilde {\Sigma }$, so that linear algebra operations can be performed using numerical methods for sparse matrices, resulting in a substantial computational gain [16].

The objective of the SPDE approach is in the way it identifies the GMRF, with local neighborhood and a sparse precision matrix, which best describes the Matérn field – a GF with a Matérn covariance function. Given this representation, it is possible to derive inference from the GMRF through the use of its good computational properties. Essentially, the SPDE approach uses a finite element representation to define the Matérn field as a linear combination of base functions defined on a triangulation of the domain $\mathcal {D}$. This consists of subdividing $\mathcal {D}$ into a set of triangles that do not intersect and have maximum one edge or vertex in common [15]. Figure 3 illustrates the concept of triangulation in SA1.

### Integrated nested Laplace approximation (INLA)

Let $\theta =\left \{\zeta,\beta,\sigma ^{2}_{\varepsilon },a,\sigma ^{2}_{\omega },\kappa \right \}$ denote the parameter vector to be estimated. The joint posterior distribution is given by:

(5) $$\begin{array}{@{}rcl@{}} \pi(\theta,\xi,\mu|{y}) \propto \pi(\,{y}|\mu)\pi(\mu|\xi,\theta)\pi(\xi|\theta)\pi(\theta)  \end{array} $$

where *π*(·) denotes the probability density function, ***y***={***y***_*t*_}, ***μ***={***μ***_*t*_} and ***ξ***={***ξ***_*t*_} with *t*=1,…,*T*. Usually, independent prior distributions are chosen for the parameters, so that $\pi (\boldsymbol {\theta })=\prod ^{\text {dim}\left (\boldsymbol {\theta }\right)}_{i=1}\pi (\boldsymbol {\theta }_{i})$. Considering that conditionally on ***μ*** the observations ***y***_*t*_ are serially independent and that the state process follows Markovian time dynamics, the Eq. (5) can be written as:

$$\begin{array}{@{}rcl@{}} \pi(\boldsymbol{\theta},\boldsymbol{\xi},\boldsymbol{\mu}|{\boldsymbol{y}}) &\propto& \left(\prod^{T}_{t=1}\pi({\boldsymbol{y}}_{t}|\boldsymbol{\mu}_{t})\right) \left(\prod^{T}_{t=1}\pi(\boldsymbol{\mu}_{t}|\boldsymbol{\xi}_{t},\boldsymbol{\theta})\right)\\&\times& \left(\pi(\boldsymbol{\xi}_{1}|\boldsymbol{\theta})\prod^{T}_{t=2}\pi(\boldsymbol{\xi}_{t} \mid \boldsymbol{\xi}_{t-1},\boldsymbol{\theta})\right)\pi(\boldsymbol{\theta})  \end{array} $$

As the distribution *π*(***θ***,***ξ***,***μ***|***y***) has no analytic solution, it is necessary to use approximation methods to sample from it. From a Bayesian perspective, the most common approach is to make inference for the model based on Markov Chain Monte Carlo (MCMC) methods [17]. However, it is possible to use the Integrated Nested Laplace Approximation (INLA) method, proposed by Rue et al. [18], as an alternative to MCMC methods. The main advantage of INLA over MCMC is computational, as the algorithm rapidly produces accurate approximations to posterior marginals distributions for the latent variables, as well as for the hyperparameters.

Unlike the MCMC, where posterior inference is based on simulations, the INLA method directly ties distributions of interest with a closed form expression. Therefore, the convergence diagnosis inherent to MCMC methods is not a problem. The main objective of the INLA approach is to approximate the posterior marginal distributions of the latent field and of the hyperparameters, given by:

(6) $$\begin{array}{@{}rcl@{}} \pi(\xi_{i}|{\boldsymbol{y}})&=&\int \pi(\xi_{i}|\boldsymbol{\theta},{\boldsymbol{y}})\pi(\boldsymbol{\theta}|{\boldsymbol{y}})d\boldsymbol{\theta} \end{array} $$

(7) $$\begin{array}{@{}rcl@{}} \pi(\theta_{j}|{\boldsymbol{y}})&=&\int \pi(\boldsymbol{\theta}|{\boldsymbol{y}})d\boldsymbol{\theta}_{-j}. \end{array} $$

This approach is based on an efficient combination of Laplace approximations for the full conditional distributions *π*(***θ***|***y***) and *π*(*ξ*_*i*_|***θ***,***y***), *i*=1,…,*n*, and numerical integration routines to integrate out the hyperparameters ***θ***.

The INLA method as proposed in Rue et al. (2009) includes three main approximation steps to obtain the marginal posteriors in (6) and (7). The first step entails approximating the full posterior *π*(***θ***|***y***). Firstly, it is necessary to obtain an approximation of the full conditional distribution of ***ξ***, *π*(***ξ***|***y***,***θ***), using a multivariate Gaussian density $\widetilde {\pi }_{G}(\boldsymbol {\xi }|\boldsymbol {y},\boldsymbol {\theta })$ (see Rue and Held [16] for further details) and evaluate it in your mode. Then, the posterior density of ***θ*** is approximated using the Laplace approximation

$$\begin{array}{@{}rcl@{}} \widetilde{\pi}(\boldsymbol{\theta}|\boldsymbol{y})\propto{\frac{\pi(\boldsymbol{\xi},\boldsymbol{\theta}, \boldsymbol{y})}{\widetilde{\pi}_{G}(\boldsymbol{\xi}|\boldsymbol{\theta},\boldsymbol{y})}} \left|\!\!\vphantom{1^{3}_{1}}\right.{}_{\boldsymbol{\xi}=\boldsymbol{\xi}^{*}\left(\boldsymbol{\theta}\right)}, \end{array} $$

where ***ξ***^∗^(***θ***) is the mode of the full conditional of ***ξ*** for a given ***θ***. Since there is no exact closed form for ***ξ***^∗^(***θ***), an optimization scheme is necessary. Rue et al. [18] calculated this mode using the Newton-Raphson algorithm. The posterior $\widetilde {\pi }(\boldsymbol {\theta }|\boldsymbol {y})$ will be used later to integrate out the uncertainty with respect to ***θ*** when approximating the posterior marginal of *ξ*_*i*_.

The second step involves calculating the Laplace approximation of the full conditionals *π*(*ξ*_*i*_|***y***,***θ***) for some values of ***θ***. These values will be used as evaluation points in the numerical integration to obtain the posterior marginals of *ξ*_*i*_ in (6). The distribution of *π*(*ξ*_*i*_|***θ***,***y***) is approximated using the Laplace approximation defined by

(8) $$\begin{array}{@{}rcl@{}} \widetilde{\pi}_{LA}\left(\xi_{i}|\boldsymbol{\theta},\boldsymbol{y}\right)\propto \frac{\pi\left(\boldsymbol{\xi},\boldsymbol{\theta},\boldsymbol{y}\right)} {\widetilde{\pi}_{G}\left(\boldsymbol{\xi}_{-i}|\xi_{i},\boldsymbol{\theta},\boldsymbol{y}\right)} \left|\!\!\vphantom{1^{3}_{1}}\right.{}_{\boldsymbol{\xi}_{-i}=\boldsymbol{\xi}^{*}_{-i}\left(\xi_{i},\boldsymbol{\theta}\right)},  \end{array} $$

where ***ξ***_−*i*_ is the vector ***ξ*** with the *i*-th component omitted, $\widetilde {\pi }_{G}\left (\boldsymbol {\xi }_{-i}|\xi _{i},\boldsymbol {\theta },\boldsymbol {y}\right)$ is the Gaussian approximation of *π*(***ξ***_−*i*_|*ξ*_*i*_,***θ***,***y***), considering *ξ*_*i*_ as fixed (observed) and $\boldsymbol {\xi }^{*}_{-i}\left (\xi _{i},\boldsymbol {\theta }\right)$ is the mode of *π*(***ξ***_−*i*_|*ξ*_*i*_,***θ***,***y***).

The approximation of *π*(*ξ*_*i*_|***θ***,***y***) using (8) can be expensive, since $\widetilde {\pi }_{G}\left (\boldsymbol {\xi }_{-i}|\xi _{i},\boldsymbol {\theta },\boldsymbol {y}\right)$ have to be recalculated for each value of *ξ*_*i*_ and ***θ***. Rue et al. [18] proposed two cheaper alternatives to obtain these distributions. The first one is the Gaussian approximation $\widetilde {\pi }_{G}\left (\xi _{i}|\boldsymbol {\theta },\boldsymbol {y}\right)$, which provides reasonable results in a short computational time; however, according to Rue and Martino [19], its accuracy can be affected by several factors. These problems can be corrected with moderate computational cost using a simplified version of the Laplace approximation, defined as the series expansion of $\widetilde {\pi }_{\textit {LA}}\left (\xi _{i}|\boldsymbol {\theta },\boldsymbol {y}\right)$ around *ξ*_*i*_=*μ*_*i*_(***θ***), the mean of $\widetilde {\pi }_{G}\left (\xi _{i}|\boldsymbol {y},\boldsymbol {\theta }\right)$ [18].

At last, the full posteriors obtained through the two previous steps are combined and the marginal densities of *ξ*_*i*_ and *θ*_*j*_ are obtained by integrating the irrelevant terms. The approximation for the marginal of the latent variables can be obtained by

(9) $$ \begin{aligned} \pi\left(\xi_{i}|\boldsymbol{y}\right) &= \int\pi\left(\xi_{i}|\boldsymbol{y},\boldsymbol{\theta}\right)\pi\left(\boldsymbol{\theta}|\boldsymbol{y}\right) d\boldsymbol{\theta}\\ &\approx\sum_{k}\widetilde{\pi}\left(\xi_{i}|\theta_{k},\boldsymbol{y}\right)\widetilde{\pi} \left(\theta_{k}|\boldsymbol{y}\right)\;\Delta_{k}, \end{aligned}  $$

which is evaluated on a set of grid points *θ*_*k*_ with weights *Δ*_*k*_, for *k*=1,2,…,*K*. According to Rue et al. [18], since the integration points are selected in a regular grid, it is feasible to assume all the weights *Δ*_*k*_ to be equal. A similar numerical integration procedure is used to evaluate the marginals *π*(*θ*_*j*_|***y***). Since the dimension of ***θ*** is small (less than or equal to seven), these numerical routines are effective in returning a discretized representation of the marginal posteriors.

A good choice of the set *θ*_*k*_ of evaluation points is important for the accuracy of the above numerical integration steps. Rue et al. [18] suggest to compute the negative Hessian matrix *S* at the mode *θ*^∗^, of $\widetilde {\pi }\left (\boldsymbol {\theta }|\boldsymbol {y}\right)$ and to consider its spectral value decomposition, *S*^−1^=*QΛQ*^*T*^. Then, ***θ*** is defined through a standardized variable *z*, such that:

$$\begin{array}{@{}rcl@{}} z = Q^{T}\Lambda^{-1/2}\left(\theta-\theta^{*}\right) \quad \text{or} \quad \theta(z)=\theta^{*}+Q\Lambda^{1/2}z \end{array} $$

and a collection *Z* of *z* values is obtained, such that the corresponding *θ*(*z*) points are located around the mode *θ*^∗^. Starting from *z*=0 (*θ*=*θ*^∗^), each component entry of *z* is searched in the positive and negative directions in step sizes of *η*_*z*_. All *z* points that satisfy

$$\begin{array}{@{}rcl@{}} \text{log}\;\widetilde{\pi}\left(\boldsymbol{\theta}(0)|\boldsymbol{y}\right)-\text{log}\;\widetilde{\pi} \left(\boldsymbol{\theta}(z)|\boldsymbol{y}\right)<\eta_{\pi} \end{array} $$

are considered to be belonging to *Z*. The set of evaluation points is based on the values in *Z*. An appropriate calibration of *η*_*z*_ and *η*_*π*_ values must be performed, in order to produce accurate approximations.

In the present work, SPDE approach was used together with the INLA method. All analyses were conducted using the R software version 3.0.1 [20] in the R-INLA package [21].

## Results

Summaries of the posterior means of the model’s fixed effects and their respective 95 % credibility intervals (CI) are shown in Table 2. The “interaction” component, whenever shown, represents the effect of the interaction between accumulated rainfall in the two weeks prior to collection and the minimum temperature in the week prior to collection on the number of eggs. In the presence of statistically significant interaction, the primary effects of variables involved in the interaction terms are not interpreted, in order to avoid erroneous conclusions. All the models fit the data reasonably well, with 58 to 88 % of the validation dataset encompassed by the 95 % CI. The validation analysis could capture the oviposition pattern for all SAs, except for the highest number of eggs. Additional file 1 shows the validation analysis per trap for one particular SA (SA6).

**Table 2:** Individual models

SA1				SA2			
Parameter	Average	CI (95 %)		Parameter	Average	CI (95 %)	
*Positivity* (*ζ*)	0.67	0.65	0.69	*Positivity* (*ζ*)	0.78	0.76	0.79
*Border distance* (*β*_1_)	0.02	–0.08	0.11	*Border distance* (*β*_1_)	**–0.11**	–0.20	–0.02
*Interaction* (*β*_4_)	**–0.69**	–1.36	–0.02	*Interaction* (*β*_4_)	—	—	—
*Rainfall* (*β*_2_)	0.80	0.13	1.47	*Rainfall* (*β*_2_)	**0.09**	0.04	0.14
*Tmin* (*β*_3_)	0.29	0.19	0.40	Tmin (*β*_3_)	**0.15**	0.07	0.24
*Temporal* (*a*)	0.40	0.32	0.47	*Temporal* (*a*)	0.46	0.38	0.52
$\textit {Nugget}\;(\sigma ^{2}_{\varepsilon })$	0.06	0.05	0.07	$\textit {Nugget}\;(\sigma ^{2}_{\varepsilon })$	0.06	0.05	0.07
$\textit {Spatial}\;(\sigma ^{2}_{\omega })$	1.45	1.30	1.62	$\textit {Spatial}\;(\sigma ^{2}_{\omega })$	1.66	1.47	1.87
*Range* (*ρ*)	25.98	23.06	29.76	*Range* (*ρ*)	21.23	18.06	24.04
SA3				SA4			
Parameter	Average	CI (95 %)		Parameter	Average	CI (95 %)	
*Positivity* (*ζ*)	0.64	0.62	0.67	Positivity (*ζ*)	0.65	0.63	0.67
*Border distance* (*β*_1_)	0.03	–0.04	0.11	*Border distance* (*β*_1_)	0.03	–0.07	0.13
*Interaction* (*β*_4_)	**0.85**	0.14	1.55	*Interaction* (*β*_4_)	—	—	—
*Rainfall* (*β*_2_)	–0.74	–1.45	–0.03	*Rainfall* (*β*_2_)	0.00	–0.05	0.06
*Tmin* (*β*_3_)	0.10	–0.00	0.19	*Tmin* (*β*_3_)	**0.12**	0.03	0.21
*Temporal* (*a*)	0.30	0.21	0.42	*Temporal* (*a*)	0.46	0.40	0.52
$\textit {Nugget}\;(\sigma ^{2}_{\varepsilon })$	0.08	0.05	0.10	$\textit {Nugget}\;(\sigma ^{2}_{\varepsilon })$	0.04	0.03	0.05
$\textit {Spatial}\;(\sigma ^{2}_{\omega })$	1.16	1.01	1.32	$\textit {Spatial}\;(\sigma ^{2}_{\omega })$	1.79	1.53	2.07
*Range* (*ρ*)	29.96	25.44	34.56	*Range* (*ρ*)	26.47	21.85	34.15
SA5				SA6			
Parameter	Average	CI (95 %)		Parameter	Average	CI (95 %)	
Positivity (*ζ*)	0.55	0.53	0.57	Positivity (*ζ*)	0.66	0.64	0.68
*Border distance* (*β*_1_)	–0.04	–0.13	0.05	*Border distance* (*β*_1_)	–0.03	–0.11	0.06
*Interaction* (*β*_4_)	—	—	—	*Interaction* (*β*_4_)	—	—	—
*Rainfall* (*β*_2_)	**0.10**	0.04	0.17	*Rainfall* (*β*_2_)	**0.11**	0.05	0.17
*Tmin* (*β*_3_)	**0.35**	0.25	0.44	*Tmin* (*β*_3_)	**0.29**	0.21	0.38
*Temporal* (*a*)	0.35	0.30	0.41	*Temporal* (*a*)	0.35	0.27	0.41
$\textit {Nugget}\;(\sigma ^{2}_{\varepsilon })$	0.09	0.07	0.10	$\textit {Nugget}\;(\sigma ^{2}_{\varepsilon })$	0.04	0.03	0.06
$\textit {Spatial}\;(\sigma ^{2}_{\omega })$	1.31	1.17	1.47	$\textit {Spatial}\;(\sigma ^{2}_{\omega })$	1.30	1.12	1.48
*Range* (*ρ*)	25.91	23.73	28.06	*Range* (*ρ*)	27.26	23.71	31.90
SA7				SA8			
Parameter	Average	CI (95 %)		Parameter	Average	CI (95 %)	
*Positivity* (*ζ*)	0.65	0.62	0.67	Positivity (*ζ*)	0.38	0.36	0.40
*Border distance* (*β*_1_)	**0.12**	0.04	0.21	*Border distance* (*β*_1_)	0.01	–0.08	0.09
*Interaction* (*β*_4_)	—	—	—	*Interaction* (*β*_4_)	—	—	—
*Rainfall* (*β*_2_)	**0.07**	0.01	0.14	*Rainfall* (*β*_2_)	**0.09**	0.02	0.16
*Tmin* (*β*_3_)	**0.30**	0.22	0.39	*Tmin* (*β*_3_)	0.06	–0.04	0.16
*Temporal* (*a*)	0.32	0.25	0.39	*Temporal* (*a*)	0.29	0.18	0.41
$\textit {Nugget}\;(\sigma ^{2}_{\varepsilon })$	0.02	0.01	0.05	$\textit {Nugget}\;(\sigma ^{2}_{\varepsilon })$	0.09	0.05	0.12
$\textit {Spatial}\;(\sigma ^{2}_{\omega })$	1.36	1.21	1.54	$\textit {Spatial}\;(\sigma ^{2}_{\omega })$	1.04	0.87	1.22
*Range* (*ρ*)	33.87	29.52	38.28	*Range* (*ρ*)	34.19	29.53	39.56

Average and 95 % credibility interval (CI) of the posterior distribution of the model’s fixed parameters in each sentinel area. Values in bold highlight the statistically significant covariates

The positivity index ranged from 0.38 to 0.78. The area with the least positivity was SA8, and the most “attractive” for oviposition was SA2. Border distance had significant effect on the reduction of the number of eggs in SA2, and contributed to an increase in eggs in SA7. Total accumulated rainfall (Rainfall) was important to explain the increase in the number of eggs in all SAs except SA4. Minimum temperature (Tmin) also significantly contributed to explain the egg frequency in all SAs, except SA8.

**Fig. 4:**
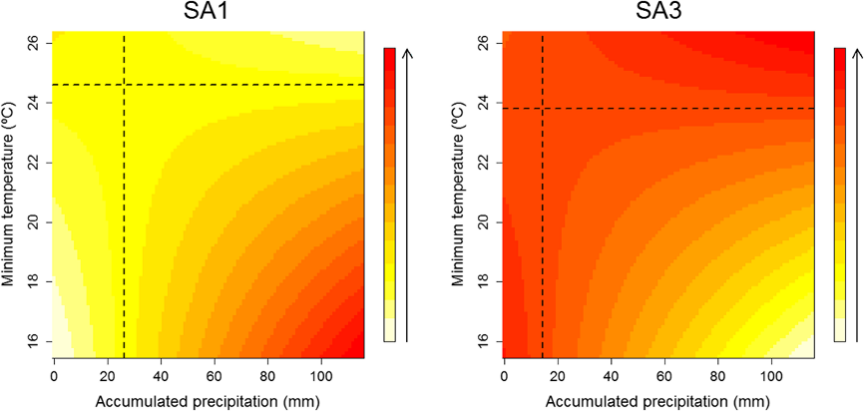
Interaction effects. Map of the interaction effects between total rainfall (lag 2) and minimum temperature (lag 1) on the average egg density in SA1 and SA3, according to changes in the total rainfall and minimum temperature. The quadrants formed by dashed black lines delimit the influence of climate variables on average egg density, according to the thresholds of 26.33 mm (total rainfall) / 24.6 °C (minimum temperature) in SA1 and 14.5 mm (total rainfall)/23.8 °C (minimum temperature) in SA3. The ascending color scale indicates the range of values for egg density with respect to the total rainfall and minimum temperature values

Interaction effects between climate variables in SA1 and SA3 suggest that the effect of the quantity of accumulated rainfall (lag 2) on egg abundance changes as a function of the minimum temperature (lag 1). Figure 4 shows how the interaction between these variables influenced egg density, highlighting the change in direction of the effects for values above or below 26.3 mm of rain and 24.6 °C minimum temperature in SA1. In this area, an increase in the number of eggs could be expected only if the total rainfall was more than 26.3 mm, and minimum temperatures fell below 24.6 °C. In SA3, the effects changed direction according to the thresholds of 14.5 mm and 23.8 °C, so that rainfall greater than 14.5 mm and temperatures below 23.8 °C favored a reduction in egg density.

The temporal correlation between the weekly average number of eggs was moderate, varying from 0.29 (SA8) to 0.46 (SA2 and SA4). The variance of the nugget effect or measurement error ranged from 0.02 (SA7) to 0.09 (SA5 and SA8). The posterior mean of the spatial effect variance ranged from 1.04 (SA8) to 1.79 (SA4). More variation is explained by the spatial term rather than by the measurement error in all SAs. The empirical range varied from 21.23 to 34.19 m (the maximum distance between traps was 182.6 and 623.8 m) occurring respectively in SA2 and SA8. As these are the distances at which correlation is close to 0.1, we can conclude that the data feature moderate spatial correlation, which slowly decreases with distance.

**Fig. 5:**
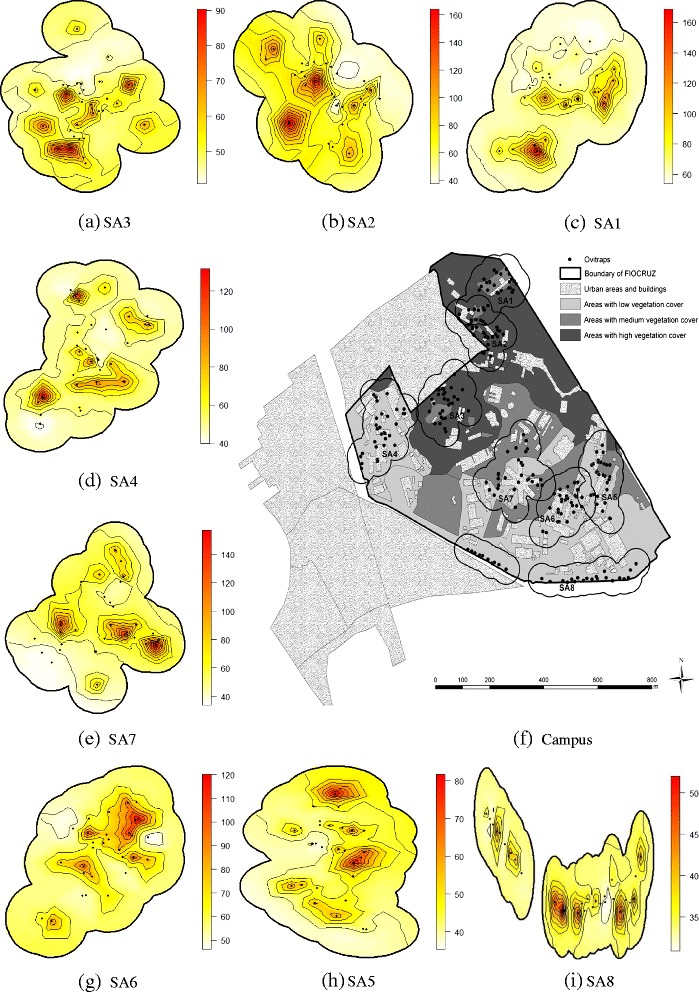
Egg frequency. Map of spatial distribution of the number of eggs in SA3 (**a**), SA2 (**b**), SA1 (**c**), SA4 (**d**), SA7 (**e**), SA6 (**g**), SA5 (**h**) and SA8 (**i**), corresponding to the weekly average per trap throughout the study period, and campus map (**f**) describing vegetation and location of ovitraps. The filled circle indicates the location of traps

The mean spatial egg distribution during the study period is presented in Fig. 5. In each area, the filled circle indicates the traps’ location. In general, the highest concentration of eggs was found in areas that bordered settlements with high population densities (slums), and in those close to campus buildings, where pedestrian foot traffic was heavier. Within each area, egg distribution was heterogeneous. Additional file 2 shows weekly changes in the number of eggs in SA1 throughout the study period using an animated map.

The hierarchical model estimated an average positivity of 62.2 %. Border distance was not important to explain the variations in the number of eggs, so it did not enter in the final model. The accumulated rainfall in the two weeks prior to trap installation was statistically significant but the minimum temperature in the previous week was not, as well as the interaction between them. As expected, higher rain rates (*β*_2_=0.08 [ 95 *%* *CI*: 0.01−0.16]) was associated with larger quantities of eggs. The variability attributed to the spatial-structured effect ($\sigma ^{2}_{\omega } = 8.98$ [ 95 *%* *CI*: 6.98−12.42]) was greater than that attributed to measurement error ($\sigma ^{2}_{\varepsilon } = 0.17$ [ 95 *%* *CI*: 0.15−0.19]), while the temporal correlation (*a*) found was 0.77 (0.71 - 0.83). The variance attributable to the random effect was 0.02 (0.02 - 0.03), suggesting homogeneity among SAs. The estimated empirical range (*ρ*=37.27 [ 95 *%* *CI*: 32.25−42.13]) suggests that the results from the hierarchical model are similar to those of individual models. The validation dataset encompassed by the 95 % CI was 57.8 %.

## Discussion

Due to the complexity of estimation involved in spatio-temporal modeling, these dimensions of variation inherent to many epidemiological processes are rarely analyzed together. The present methodology for estimation allows for the rapid and efficient implementation of these models, while considering 1) zero-inflation within SA, 2) interaction among climate variables and 3) different oviposition patterns over the course of the study period. This methodology can contribute to the identification of priority areas for entomological surveillance and targeted fieldwork.

Oviposition activity occurred over the entire course of the study but varied among SAs. The highest ovitrap positivity was 78 % (76–79 %) in SA2, while the lowest one was 38 % (36–38 %) in SA8. The remaining SAs presented ovitrap positivities varying between 55 and 67 % (Table 2). As expected, summertime featured the greatest infestation.

The distance between ovitraps and the border of the campus was assessed based on the hypothesis that mosquitoes came from outside the campus [8]. If this was true, significant negative effect would be found. However, such result was only observed in SA2, which limits a densely populated slum. All the other SAs presented nonsignificant or inverted relationship (SA7). This opposite trend suggests that the eggs captured in SA7 are not from outside mosquitoes, but from mosquitoes established inside. Within the campus, the greatest egg collection occurred in areas close to buildings and passages, in comparison to more isolated areas. Indeed, on the same campus, Honório et al. [8] found statistically significant relationship between the number of mosquito larvae in artificial breeding sites within the campus and the distance to the border in both the wet and dry seasons. The anthropophilic behavior of *Aedes aegypti* is well documented.

A model containing the interaction between temperature and rain was more informative and provides thresholds that could be used to issue alerts. However, the threshold values differed between areas, being significant only in two areas, SA1 and SA3 (Fig. 4). This variation suggests local microclimate effects not captured by the single meteorological station and can be attributed to the particular characteristics of each area (Table 1). Minh An and Rocklöv [22] evaluated the effect of the interaction between rain and temperature on the number of dengue cases in Hanoi, Vietnam. They showed rain occurrence and temperatures between 15 and 30 °C to correlate to an increase in the number of dengue cases.

Total precipitation accumulated over the two weeks prior to collection contributed to the increase in the number of eggs in five of the SAs, located within both high and low levels of vegetation areas. SA1 and SA3 also featured the effect of rain, but this effect interacted with temperature. Only SA4 showed no association between the number of eggs and rainfall. The relationship between precipitation and the proliferation of *A. aegypti* is likely to vary on small geographical scales [23]. Rain contributes to the formation of breeding habitats, but this effect will depend on the local availability of containers [24]. During heavy rainfall, these water containers offer favorable conditions for oviposition and the development of immature mosquitoes.

Significant positive relationship between minimum temperature and the number of eggs deposited over the following week was found in almost all SAs. High temperatures increase the rate at which mosquito larvae develop, leading to the rapid subsequent development of adult life forms of the mosquito. Under such conditions, the frequency of mosquito bites in humans also increases [25, 26]. Honório et al. [27] also found a significant positive effect of temperature on *A. aegypti* egg density in three neighborhoods of the municipality of Rio de Janeiro.

In the present study, we identified spatial patterns consistent with human population in all sentinel areas. Duncombe et al. [28] showed that vector density tends to correlate directly to high population density. A Colombian study also reinforced these findings [29]. The presence or absence of spatial correlation could be influenced by the distance between traps. Our findings suggest that ideal entomological surveillance should occur with weekly visits to traps located between 18 and 24 m apart. This result is important to guide the implementation of ovitrap surveillance systems, but also presents a challenge due to the high sampling effort required. Often surveillance is carried out using more sparsely distributed traps, from 50 m to 200 m apart [7]. One possibility to overcome this problem is to cluster traps in sentinel areas, as done in this study.

The joint analysis of the sentinel areas (Hierarchical model) only confirmed the rainfall as driving the number of eggs. Nevertheless, the estimates of spatial and temporal dependence parameters were similar.

The present study had some limitations. Other data, such as wind direction and speed were not available for analysis, as well as other potentially confounding variables, such as the amount and location of breeding sites. Moreover, precipitation data were collected from a weather station 5 km away from the study area. This may introduce bias, as the quantity of rainfall can vary substantially even in close geographical areas.

Aside from these limitations, the results suggest that border distance, minimum temperature and precipitation are all associated with population density of *A. aegypti*. Maps describing the abundance of eggs identified areas with high potential for transmission, so that control and prevention activities could be developed. The results also indicates the ideal spacing for traps, which constitutes an important aspect of sampling.

## Conclusions

*Aedes aegypti*, due to it short generation time and strong response to climate triggers, tend to show an eruptive dynamics that is difficult to predict and understand through just temporal or spatial models. Spatio-temporal modeling has been prohibitive due the computational costs involved in MCMC based parameter estimation. Our results suggest that INLA based inference increases the efficiency of the estimation process in a way to allow its calculation within the time frame expected for any surveillance program. Differently from other ovitrap surveys, the studied dataset consisted of a high density of traps placed at close distances, and surveyed very frequently. This design allowed to assess the spatial and temporal autocorrelation structure of the oviposition process. The short range of these correlations support the notion that high sampling is necessary to capture the spatio-temporal patterns of mosquito activity, for example, the identification of hotspots. The extrapolation of these results to other areas must be done with care. Ideally, this study should be replicated in other settings. However, despite the specific results obtained, we believe this framework (zero-inflation + truncated Poisson model + INLA based inference) is an efficient way to model oviposition dynamics anywhere.

## Additional files

Additional file 1 **Validation analysis for SA6.** Validation analysis per trap for SA6 throughout the study period. (PDF 23.6 Kb)

Additional file 2 **Animated map for SA1.** Animated map of weekly changes in the number of eggs in SA1 throughout the study period. This file can be viewed with: QuickTime Player. (MP4 11980 kb)

## Abbreviations

BI: Breteau index
Bin: Binomial distribution
CI: Credibility intervals
DIC: Deviance information criterion
Fiocruz: Oswaldo Cruz foundation
GF: Gaussian field
GMRF: Gaussian Markov random field
HI: Household infestation index
ICICT: Institute of scientific and technological communication and information in health
INLA: Integrated nested Laplace approximation
MCMC: Markov chain Monte Carlo methods
NOSMOVE: Sentinel operational unit of mosquito vectors
Rainfall: Total accumulated rainfall
SA: Sentinel areas
SPDE: Stochastic partial differential equations
Tmin: Minimum temperature
VPAAPS: Vice-presidency of environment, healthcare and health promotion
ZAP: Zero-altered poisson distribution
