# Supplementary figures and images for: Surveillance of dengue vectors using spatio-temporal Bayesian modeling
Source: BMC Med Inform Decis Mak. 2015 Nov 13;15:93. doi: 10.1186/s12911-015-0219-6 (PMC4644323; doi:10.1186/s12911-015-0219-6)

SA6 - trap 1

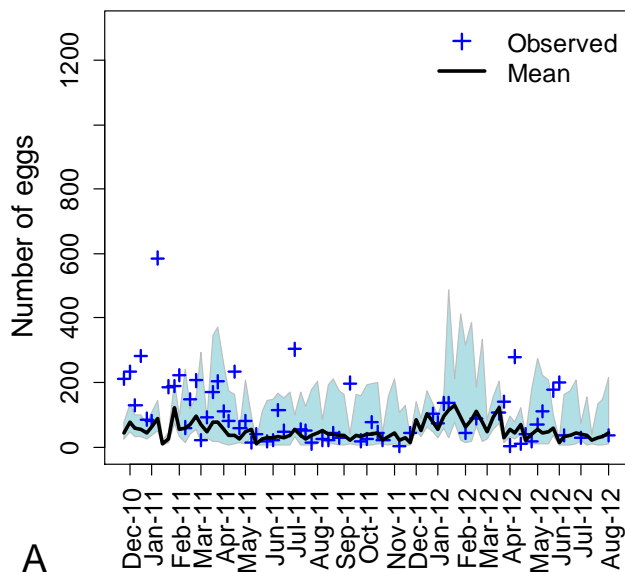

A

SA6 - trap 2

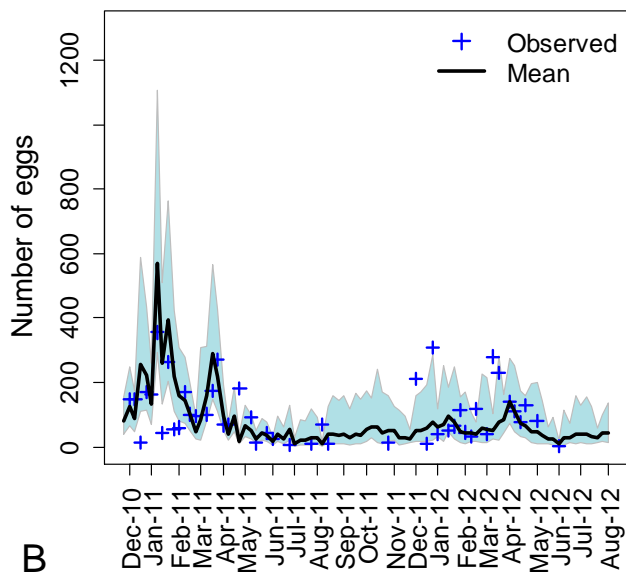

B

SA6 - trap 3

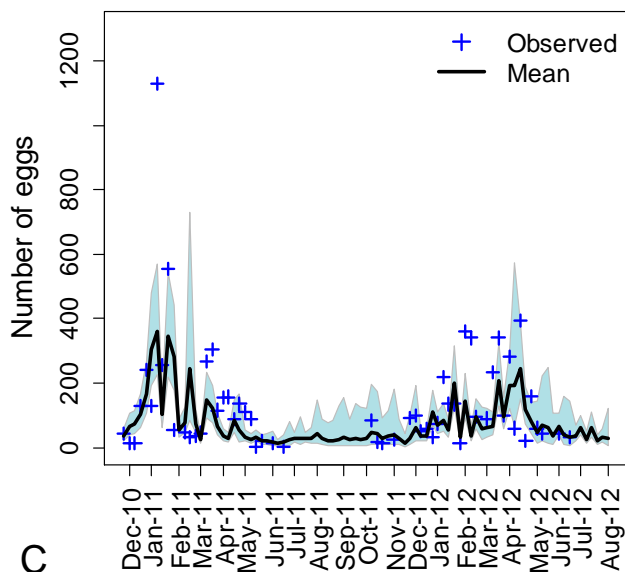

C

SA6 - trap 4

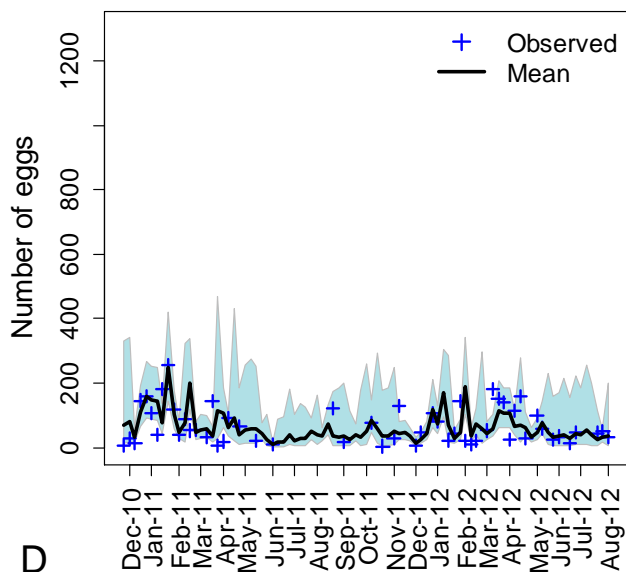

D

SA6 - trap 5

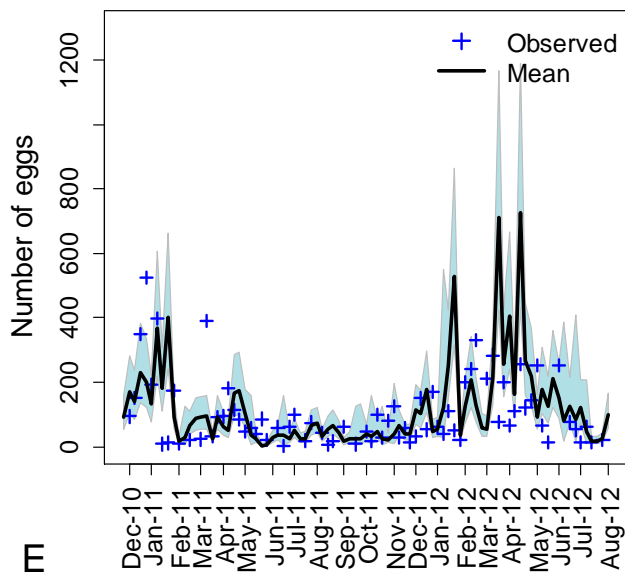

E

SA6 - trap 6

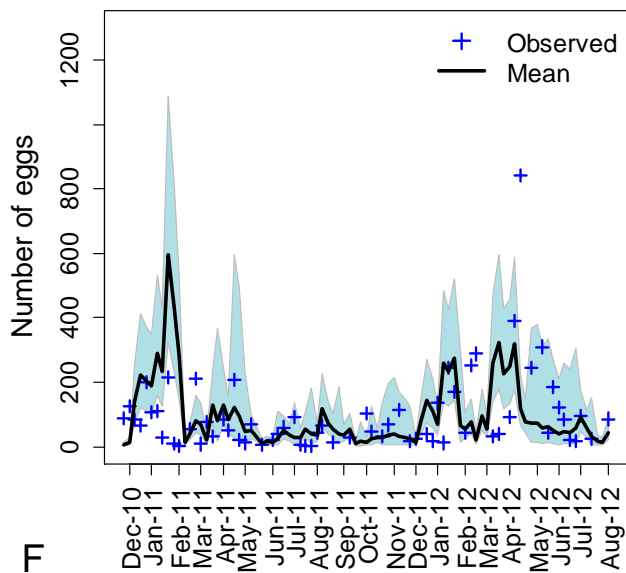

F

Supplement: Supplementary file 1 — Validation analysis for SA6. Validation analysis per trap for SA6 throughout the study period. (PDF 23.6 Kb) [file 12911_2015_219_MOESM1_ESM.pdf]
